# Supplementary material for: Lamivudine and Emtricitabine Dosing Proposal for Children with HIV and Chronic Kidney Disease, Supported by Physiologically Based Pharmacokinetic Modelling
Source: Pharmaceutics. 2023 May 6;15(5):1424. doi: 10.3390/pharmaceutics15051424 (PMC10221211; doi:10.3390/pharmaceutics15051424)
Supplement: Supplementary file 1 [file pharmaceutics-15-01424-s001.zip › pharmaceutics-2346223-supplementary.pdf]

Supplementary File

Lamivudine and Emtricitabine Dosing Proposal for Children with HIV and Renal Impairment, Supported by Physiologically-based Pharmacokinetic Modelling

Tom G. Jacobs, Marika A. de Hoop-Sommen, Thomas Nieuwenstein, Joyce E. M. van der Heijden, Saskia N. de Wildt, David M. Burger, Angela Colbers, Jolien J. M. Freriksen

Table of contents

General

Table S1. General input parameters compound models 3TC, FTC and GCV

Figure S1. Types of model input parameter adjustments

Verification simulations

Table S2. Model input parameters for 3TC

Table S3. Model input parameters for FTC

Table S4. Model input parameters for GCV

Table S5. Model input parameters for GCV in paediatrics

Table S6. Model input parameters for 3TC and FTC in paediatrics

Table S7. Model verification: prediction errors

Table S8. Model verification: individual GCV prediction errors in paediatric populations

Figure S2. Model verification: visual predictive checks

S2.1. Visual predictive checks for prediction of 3TC PK in adults with various degrees of CKD.

S2.2 Visual predictive checks for prediction of 3TC PK in children with normal kidney function.

S2.3 Visual predictive checks for prediction of FTC PK in adults with various degrees of CKD.

S2.4 Visual predictive checks for prediction of FTC PK in children with normal kidney function.

Prospective simulations (3TC and FTC in paediatric subjects with CKD)

Table S9. Simulation results for 3TC PK in non-CKD and CKD populations

Table S10. Simulation results for FTC PK in non-CKD and CKD populations

Table S1. General input parameters compound models 3TC, FTC and GCV.

	Parameter	3TC[1]	FTC[2]	GCV[3]
Physicochemical properties	Molecular weight (g/mol)	229.26	247.0	255.23
	Log P	-1.40	-0.43	1.66
	Compound type	MB	MB	Ampholyte
	pKa	4.23	2.65	2.2; 9.400
	B/P	0.61	1	1
	fu	0.99	0.96	0.98
	Plasma binding protein	HSA	AGP	HSA
Absorption	fa	0.80	0.93*	0.61
	ka (1/h)	0.72	0.54	2.56
	fu_{gut}	1	1	0.1554 (P)
	Q_{gut} (L/h)	8.3472 (P)	11.816 (P)	15.601 (P)
	$P_{eff,man}$ (10^{-4}cm/s)	1.47	2.7162 (P)	5.3786 (P)
	$P_{trans,0}$ (10^{-6} cm/s)	n/a	n/a	117.91 (P)
	Permeability Assay; values	n/a	Caco-2 (7.4:7.4); 25 (P); Propranolol	n/a
Distribution	Distribution model	Full	Full	Full
	V_{SS} (L/kg)	0.49608 (P)	0.51631 (P)	0.70603 (P)
	Prediction method	Method 2	Method 2	Method 2
Transport (kidney)	Mech KiM	Yes	Yes	Yes
	CL_{PD} (mL/min/million proximal tubular cells)	4.26E-07	0	0
	Transporters, basal/apical, CLint (mL/min)	SLC22A2 (OCT2), basal, 7.4	SLC22A2 (OCT2), basal, 7.18	SLC22A6 (OAT1), basal, 11.3
		SLC47As (MATEs), apical, 3.6	ABCC4 (MRP4), apical, 1	SLC47As (MATEs), apical, 1
Elimination	Enzymes	n/a	n/a	n/a
	CL_R (L/h)	19.2	13	12.2
	Additional systemic CL (L/h)	6.1	5.1	0

Parameter values are obtained from the compound models published by Shah et al., Mendes et al., and Hsueh et al.[1-3].

Abbreviations: AGP, alpha-1 acid glycoprotein; B/P, blood-to-plasma partition ratio; CL_{int}, *in vitro* intrinsic clearance; CL_{PD}, passive diffusion clearance; CL_R, typical renal clearance for a 20-30 year healthy male; fa, fraction absorbed; fu_{gut}, unbound fraction of drug in enterocytes; HSA, human serum albumin; ka, first order absorption rate constant; Log P, buffer partition coefficient; MATE, multidrug and toxic compound extrusion; MB, monoprotic base; Mech KiM, permeability-limited mechanistic kidney model; MRP4, multidrug resistance protein 4; OAT1, organic anion transporter 1; OCT2, organic cation transporter 2; $P_{eff,man}$, human jejunum effective permeability; (P), predicted; $P_{trans,0}$, intrinsic transcellular permeability; pKa, acid dissociation constant; Q_{gut}, gut flow; V_{SS}, volume of distribution at steady state (healthy adult population). n/a: not applicable.

Figure S1. Types of model input parameter adjustments.

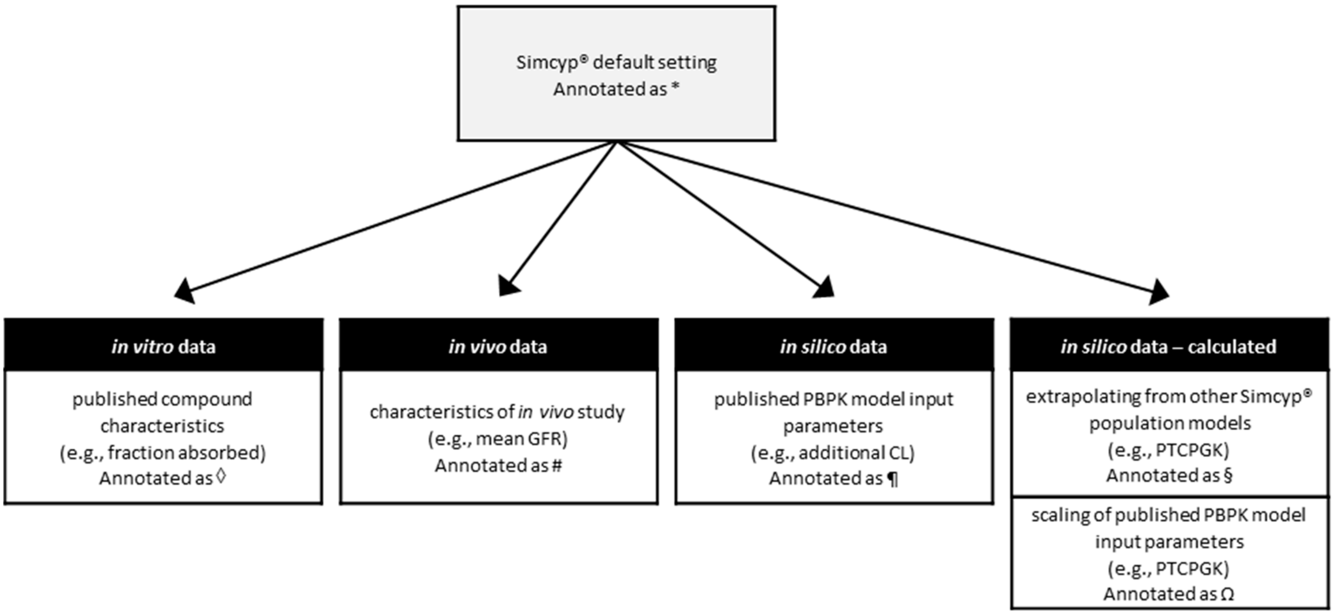

Table S2. Model input parameters for 3TC.

Parameters	POPULATIONS					
	Adults					Children with normal kidney function
	Normal kidney function	CKD G3 (Johnson)	CKD G3 (Heald)	CKD G4 (Johnson)	CKD G4 (Heald)	
GFR range ($mL/min/1.73m^2$)	>90	30-50[#]	10-40[##]	15-30[#]	<10[##]	>90*
Mean GFR ($mL/min/1.73m^2$)	127[##]	40[#]	26.9[##]	22.5[#]	6.7[##]	119
Kidney size baseline	15.4	8.4	8.4	5.7	5.7	-
Body weight coefficient	2.04	1.64	1.64	1.04	1.04	-
Body height coefficient	51.8	32.8	32.8	29.8	29.8	-
PTCPGK (cells/g)	60	26[Ω]	20.9[¶]	19.2[Ω]	9.29[¶]	60
COMPOUND MODEL 3TC						
CL_{add} (mL/min)	6.1[¶]	3.14[Ω]	2.7[¶]	2.55[Ω]	0.53[¶]	6.1[¶]

Simcyp default settings, unless otherwise specified:

* It should be noted that children having GFR <60 $mL/min/1.73m^2$ were excluded from the clinical study, though the specific values of their GFR were not reported in the paper[4].

According to the characteristics of the *in vivo* study population of Johnson et al.[5]

According to the characteristics of the *in vivo* study population of Heald et al.[6]

¶ According to the PBPK model of Shah et al.[1]

Ω Based on linear scaling by plotting mean GFR of the healthy population (127 $mL/min/1.73m^2$) and CKD G3 population (26.9 $mL/min/1.73m^2$) against values of parameter of interest (PTCPGK or CL_{add}) as applied in the PBPK model by Shah et al.[1]. This resulted in the following linear regression equations (x = mean GFR in $mL/min/1.73m^2$, PTCPGK in cells/g and additional clearance in mL/min):

- $PTCPGK = 0.3906x + 10.393$
- $CL_{add} = 0.034x + 1.7863$

Abbreviations: 3TC, lamivudine; CL_{add}, additional clearance; fa, fraction absorbed; G, grade; GFR, glomerular filtration rate; PTCPGK, proximal tubule cells per gram of kidney

Table S3. Model input parameters for FTC.

Parameters	POPULATIONS				
	Adults			Children	
	Normal kidney function	CKD G3	CKD G4	Normal kidney function (solution)	Normal kidney function (tablets)
GFR range (mL/min/1.73m^2)	>90	30-50#	15-30	>90	>90
Mean GFR (mL/min/1.73m^2)	137.5##	40#	22.5	119	119
Kidney size baseline	15.4	8.4	5.7	-	-
Body weight coefficient	2.04	1.64	1.04	-	-
Body height coefficient	51.8	32.8	29.8	-	-
PTCPGK (cells/g)	60	26§	19.2§	60	60
**COMPOUND MODEL FTC**					
CL_{add} (mL/min)	5.0¶	2.575§§	2.09§§	5.0¶	5.0¶
fa	0.93¶			0.75◊	0.93◊

Simcyp default settings, unless otherwise specified:
According to the characteristics of the *in vivo* population or CKD studies included in the Emtriva biopharmaceutical review of the FDA[7].
According to the characteristics of the *in vivo* study population of Wang et al.[8].
¶ Based on the compound model created by Mendes et al.[2]
◊ Based on the bioavailability study reported in Emtriva FDA biopharmaceutical review and the *in vivo* study of Wang et al.[7,9]
§ Based on PTCPGK value as incorporated in the published 3TC PBPK model by Shah et al.[1]. Parameter value was calculated based on linear scaling as described in Table S3 and assumed to be similar for 3TC and FTC.
§§ Based on the CL_{add} value as incorporated in the published 3TC PBPK model by Mendes et al.[2] FTC CL_{add} for CKD populations was calculated using a similar proportional reduction as applied for 3TC in CKD populations.

Abbreviations: CL_{add}, additional clearance; fa, fraction of a dose absorbed; FTC, emtricitabine; G, grade; GFR, glomerular filtration rate; PTCPGK, proximal tubule cells per gram of kidney

Table S4. Model input parameters for GCV in adults.

Parameters	POPULATIONS				
	Adults				Children with normal kidney function
	Normal kidney function	CKD G2	CKD G3	CKD G4	
GFR range (mL/min/1.73m^2)	>90	60-90	30-60	15-30	>90
Mean GFR (mL/min/1.73m^2)	99#	74#	40#	15#	119
Kidney size baseline	15.4	15.4	8.4	5.7	-
Body weight coefficient	2.04	2.04	1.64	1.04	-
Body height coefficient	51.8	51.8	32.8	29.8	-
PTCPGK (cells/g)	60	33.6¶	32.3¶	11.0¶	60
**COMPOUND MODEL GCV**					
fa	0.61¶				

Simcyp default settings, unless otherwise specified:

According to the characteristics of the *in vivo* study population of Czock et al.[10]

¶ According to the PBPK model by Hsueh et al.[3].

Abbreviations: fa, fraction of a dose absorbed; GCV, ganciclovir; CKD, chronic kidney disease; G, grade; GFR, glomerular filtration rate; PTCPGK, proximal tubule cells per gram of kidney.

Table S5. Model input parameters for GCV in paediatrics.

Parameters	PAEDIATRIC POPULATIONS		
	**Normal kidney function**	**CKD G2**	**CKD G3**
GFR range (mL/min/1.73m^2)	>90	60-90	30-60
GFR (mL/min/1.73m^2)	Fixed#	Fixed#	Fixed#
Body weight coefficient ratio	-	-	0.8§
PTCPGK (cells/g)	60	33.6¶	32.3¶
**COMPOUND MODEL GCV**			
fa	0.61¶		

Note that no simulations were performed of GCV PK in paediatrics with CKD G4.

Simcyp default settings, unless otherwise specified:

GFR was fixed at the actual GFR of the clinical subjects individually based on the characteristics of the *in vivo* study population of Vaudry et al.[11]

¶ According to the PBPK model by Hsueh et al.[3]

§ The equation used to calculate the kidney volume in an adult population including body weight and height coefficients differs from the equation used in paediatric populations. We have applied the same ratio with respect to body weight for the paediatric CKD populations as applied to adults (e.g. CKD G3 / healthy for body weight coefficient: 1.64/2.04 = 0.80) and incorporated these ratios in the equation for calculation of paediatric kidney volume. The equations were:

- Normal kidney function and CKD G2 : $\text{Kidney volume} = \frac{\left(4.214 * WT^{0.823} + 4.456 * WT^{0.795}\right)}{1000}$
- CKD G3: $\text{Kidney volume} = \frac{\left(4.214 * 0.80 * WT^{0.823} + 4.456 * 0.80 * WT^{0.795}\right)}{1000}$

where WT = weight.

Abbreviations: fa, fraction of a dose absorbed; GCV, ganciclovir; CKD, chronic kidney disease; G, grade; GFR, glomerular filtration rate; PTCPGK, proximal tubule cells per gram of kidney.

Table S6. Model input parameters for 3TC and FTC in paediatrics.

Parameters	PAEDIATRIC POPULATIONS		
	Normal kidney function	CKD G3	CKD G4
GFR range ($mL/min/1.73m^2$)	>90	30-50	15-30
GFR scaling factor	-	0.375§	0.21§
Body weight coefficient ratio	-	0.8§§	0.51§§
PTCPGK (cells/g)	60	26§§§	19.2§§§
**COMPOUND MODEL 3TC**			
fa	0.8¶		
CL_{add}(mL/min)	6.1¶	3.14§§§	2.55§§§
**COMPOUND MODEL FTC**			
fa tablet	0.93¶¶		
fa solution	0.75◊		
CL_{add} (mL/min)	5.0¶¶	2.575§§§	2.09§§§

Simcyp default settings, unless otherwise specified:
§ A GFR scaling factor was determined to achieve a predicted mean GFR within the range of the corresponding CKD group. The GFR scaling factor was multiplied by the validated Simcyp function as a 'user-defined kidney function'. In order to establish the most appropriate scaling factor, various populations were simulated with different scaling factors. The population with the most GFRs within the reference range was selected as the optimal scaling factor:

- CKD G3 : $\mathrm{GFR} = 0.375 * (-17.74 + 99.054 * \mathrm{BSA} - 6.1604 * \mathrm{BSA}^2)$
- CKD G4: $\mathrm{GFR} = 0.21 * (-17.74 + 99.054 * \mathrm{BSA} - 6.1604 * \mathrm{BSA}^2)$

CV% was unchanged (set at 15%) to include intersubject variability in kidney function and ensure that the range of GFR values was covered in the virtual CKD populations.
§§ The equation used to calculate the kidney volume in an adult population including body weight and height coefficients differs from the equation used in paediatric populations. We have applied the same ratio with respect to body weight for the paediatric CKD populations as applied to adults (e.g.ie. CKD G3 / normal kidney function: 1.64 / 2.04 = 0.80) and incorporated these ratios in the equation for calculation of paediatric kidney volume. The equations were:

- Normal kidney function: $\text{Kidney volume} = \frac{(4.214 * \mathrm{WT}^{0.823} + 4.456 * \mathrm{WT}^{0.795})}{1000}$
- CKD G3: $\text{Kidney volume} = \frac{(4.214 * 0.80 * \mathrm{WT}^{0.823} + 4.456 * 0.80 * \mathrm{WT}^{0.795})}{1000}$
- CKD G4: $\text{Kidney volume} = \frac{(4.214 * 0.51 * \mathrm{WT}^{0.823} + 4.456 * 0.51 * \mathrm{WT}^{0.795})}{1000}$

where WT = weight.
§§§ Extrapolated directly from 3TC and FTC compound models for adult CKD population models (described in more detail in Table S3 and S4).
¶ Based on the 3TC compound model created by Shah et al. [1].

¶¶ Based on the FTC compound model created by Mendes et al. [2].
◊ Based on the bioavailability study reported in Emtriva FDA biopharmaceutical review and the *in vivo* study of Wang et al.[7,9]

Of note, no simulations were conducted for the paediatric population with CKD G2 (GFR 60-90 mL/min/1.73m^2) as there are no clinical data available to verify the PBPK model for 3TC or FTC in the adult population with CKD G2.

Abbreviations: 3TC, lamivudine; BSA, body surface area; CL_{add}, additional clearance; CV%, coefficient of variation; fa, fraction absorbed; FTC, emtricitabine; GFR, glomerular filtration rate; PTCPGK, proximal tubule cells per gram of kidney.

Table S7. Model verification: prediction errors.

Drug	Population		Reference	Number of participants	Age range (years)	Female/ male	Dose	Study type SD/MD	Study duration (τ)	Prediction error $AUC_{0-\tau}$	Prediction error C_{max}	Prediction error CL/F
3TC	Adults	HIV; no CKD	Heald et al.[6]	6	29 – 41	0/6	300 mg	SD	48h	1.13	0.88	1.19
		Healthy; no CKD	Johnson et al.[5]	9	23 – 64	-	300 mg	SD	24h	0.82	0.67	1.88
		HIV; GFR 10-40 mL/min/1.73m^2	Heald et al.[6]	4	29 – 68	1/3	300 mg	SD	48h	0.74	1.02	1.85
		Healthy; GFR 30-50 mL/min/1.73m^2	Johnson et al.[5]	8	40 – 65	-	100 mg	SD	24h	0.74	0.72	1.85
		HIV; GFR <10 mL/min/1.73m^2	Heald et al.[6]	6	31 – 51	1/5	300 mg	SD	48h	1.04	0.89	0.91
		Healthy; GFR 10-30 mL/min/1.73m^2	Johnson et al.[5]	6	24 – 63	-	100 mg	SD	24h	1.21	1.17	1.01
	Paediatrics	HIV; ≤25kg	Burger et al.[4]	13	1.7 – 10	10/3	4 mg/kg	MD	12h	1.19	1.02	1.31
		HIV; >25kg	Burger et al.[4]	7	7.1 – 18	3/4	150 mg	MD	12h	1.10	0.89	1.37
FTC	Adults	Healthy; no CKD	FDA review[7]	6	-	0/6	200 mg	SD	24h	0.99	0.81	1.09
		HIV; GFR 30-50 mL/min/1.73m^2	FDA review[7]	6	54 – 78	1/5	200 mg	MD	48h	1.11	0.81	0.88
		HIV; GFR 15-30 mL/min/1.73m^2	FDA review[7]	5	48 – 78	0/5	200 mg	MD	72h	1.32	1.03	0.77
	Paediatrics	HIV; <2 years	Wang et al.[9]	2	1.8 – 1.9	0/2	120 mg/m^2 solution	SD	24h	1.30	1.36	0.81

		HIV; 2-6 years	Wang et al.[9]	8	2.3 – 5.7	3/5	120 mg/m^2 solution	SD	24h	1.39	1.07	0.72
		HIV; 6-12 years	Wang et al.[9]	8	6.6 – 10.7	4/4	120 mg/m^2 solution	SD	24h	1.34	1.01	0.74
		HIV; 6-11 years	Gaur et al.[12]	50	6.0 – 11.9	32/18	200 mg DT	MD	24h	1.17	0.96	N/A
		HIV; 12-17 years	Gaur et al.[12]	50	12 – 17.9	27/23	200 mg DT	MD	24h	0.99	0.88	N/A
GCV	Adults	Healthy; no CKD	Czock et al.[10]	12	40 ± 13	5/7	900 mg	SD	24h	0.98	1.17	1.15
		Healthy; GFR 60-90 mL/min/1.73m^2	Czock et al.[10]	6	60 ± 5	3/3	900 mg	SD	36h	0.81	1.13	1.22
		Healthy; GFR 30-60 mL/min/1.73m^2	Czock et al.[10]	6	46 ± 20	0/6	900 mg	SD	48h	0.69	1.15	1.40
		Healthy; GFR 15-30 mL/min/1.73m^2	Czock et al.[10]	6	52 ± 5	0/6	900 mg	SD	72h	0.78	1.02	1.41
	Paediatrics	Transplant recipients; no CKD	Vaudry et al.[11]	23	0.2 – 14.9	-	dose (mg) = 7 x BSA x GFR	MD	24h	0.89	N/A	N/A
		Transplant recipients; GFR 60-90 mL/min/1.73m^2	Vaudry et al.[11]	10	0.5 – 14.9	-	dose (mg) = 7 x BSA x GFR	MD	24h	0.89	N/A	N/A
		Transplant recipients; CKD G3	Vaudry et al.[11]		0.25 – 14.9	-	dose (mg) = 7 x BSA x GFR	MD	24h	1.14	N/A	N/A

		GFR 30-60 mL/min/1.73m^2										

Prediction error is calculated as predicted/observed PK parameter value. Note that the observed data are derived from patients living with HIV for some populations, while HIV-related pathophysiology is not taken into account in the model (i.e. simulations are conducted in healthy virtual subjects). N/A: observed data not available.
Abbreviations: 3TC, lamivudine; BSA, body surface area; CKD, chronic kidney disease; DT, dispersible tablet; FTC, emtricitabine; GCV, ganciclovir; GFR, glomerular filtration rate; HIV, human immunodeficiency virus; MD, multiple dose; SD, single dose.

Table S8. Model verification: individual GCV prediction errors in paediatric populations.

Subject	GCV equivalent dose (mg)	BSA (m^2)	GFR (mL/min)	BSA-adjusted GFR ($mL/min/1.73m^2$)	CKD group	Observed AUC (h·mg/L)	Predicted AUC (h·mg/L)	Prediction error
1	196.6	1.14	22.54	34.15	CKD G3	40.99	32.45	0.79
2	343.7	1.63	39.40	41.73	CKD G3	64.44	36.88	0.57
3	75.4	0.33	8.64	45.26	CKD G3	33.72	57.39	1.70
4	301.9	1.15	34.61	52.03	CKD G3	44.51	45.50	1.02
5	473.5	1.75	54.29	53.66	CKD G3	22.52	41.69	1.85
6	334.9	1.20	38.40	55.28	CKD G3	51.88	46.64	0.90
7	327.0	1.07	37.49	60.43	CKD G2	63.50	43.64	0.69
8	174.0	0.55	19.95	62.60	CKD G2	76.99	49.73	0.65
9	566.3	1.74	64.93	64.50	CKD G2	40.36	44.71	1.11
10	154.2	0.41	17.68	74.53	CKD G2	65.27	57.87	0.89
11	140.5	0.37	16.11	74.80	CKD G2	86.85	59.62	0.69
12	151.0	0.38	17.31	78.59	CKD G2	41.50	60.05	1.45
13	198.8	0.49	22.79	80.22	CKD G2	60.39	56.11	0.93
14	465.3	1.13	53.34	81.57	CKD G2	80.62	49.49	0.61
15	643.0	1.47	73.71	86.72	CKD G2	39.22	49.29	1.26
16	172.2	0.39	19.74	87.80	CKD G2	98.26	61.58	0.63
17	153.6	0.33	17.61	92.14	No CKD	41.50	56.79	1.37
18	648.2	1.49	81.85	94.85	No CKD	92.97	36.52	0.39
19	410.1	0.85	47.02	95.39	No CKD	49.39	43.47	0.88
20	176.3	0.36	20.22	96.75	No CKD	62.36	55.98	0.90
21	648.2	1.36	77.69	98.64	No CKD	72.11	39.72	0.55
22	274.4	0.52	31.46	104.61	No CKD	45.24	50.53	1.12
23	316.1	0.53	36.24	117.62	No CKD	59.87	53.09	0.89

24	257.4	0.43	29.51	118.70	No CKD	53.75	56.58	1.05
25	332.5	0.54	38.12	121.68	No CKD	46.38	53.34	1.15
26	648.2	1.45	111.94	133.33	No CKD	33.62	31.78	0.95
27	219.2	0.32	25.13	135.23	No CKD	34.03	66.10	1.94
28	431.4	0.63	49.46	136.04	No CKD	73.88	53.83	0.73
29	648.2	1.56	124.95	138.48	No CKD	42.44	28.75	0.68
30	546.1	0.77	62.61	140.38	No CKD	64.23	52.79	0.82
31	260.5	0.35	29.86	146.61	No CKD	52.30	64.98	1.24
32	599.7	0.78	68.76	152.03	No CKD	59.35	54.08	0.91
33	333.9	0.42	38.28	156.64	No CKD	53.85	62.92	1.17
34	648.2	1.44	137.08	164.50	No CKD	37.56	28.41	0.76
35	312.1	0.36	35.78	170.73	No CKD	152.43	67.98	0.45
36	648.2	1.43	141.91	171.27	No CKD	107.50	27.92	0.26
37	472.1	0.54	54.12	172.63	No CKD	81.45	60.55	0.74
38	648.2	1.54	161.92	181.57	No CKD	37.56	24.94	0.66
39	648.2	0.61	89.37	253.12	No CKD	65.58	55.18	0.84

Of note, the dose of valganciclovir (molecular weight: 354.362 g/mol) was first converted to its equivalent GCV dose based on differences in molecular weight (e.g. 900 mg valganciclovir equals 648.23 mg GCV; 900 * (255.23/354.362) = 648.23).

Abbreviations: AUC, area-under-curve; BSA, body surface area; fa, fraction of a dose absorbed; GCV, ganciclovir; CKD, chronic kidney disease; GFR, glomerular filtration rate.

Figure S2. Model verification: visual predictive checks.

Figure S2.1. Visual predictive checks for prediction of 3TC PK in adults with various degrees of CKD.

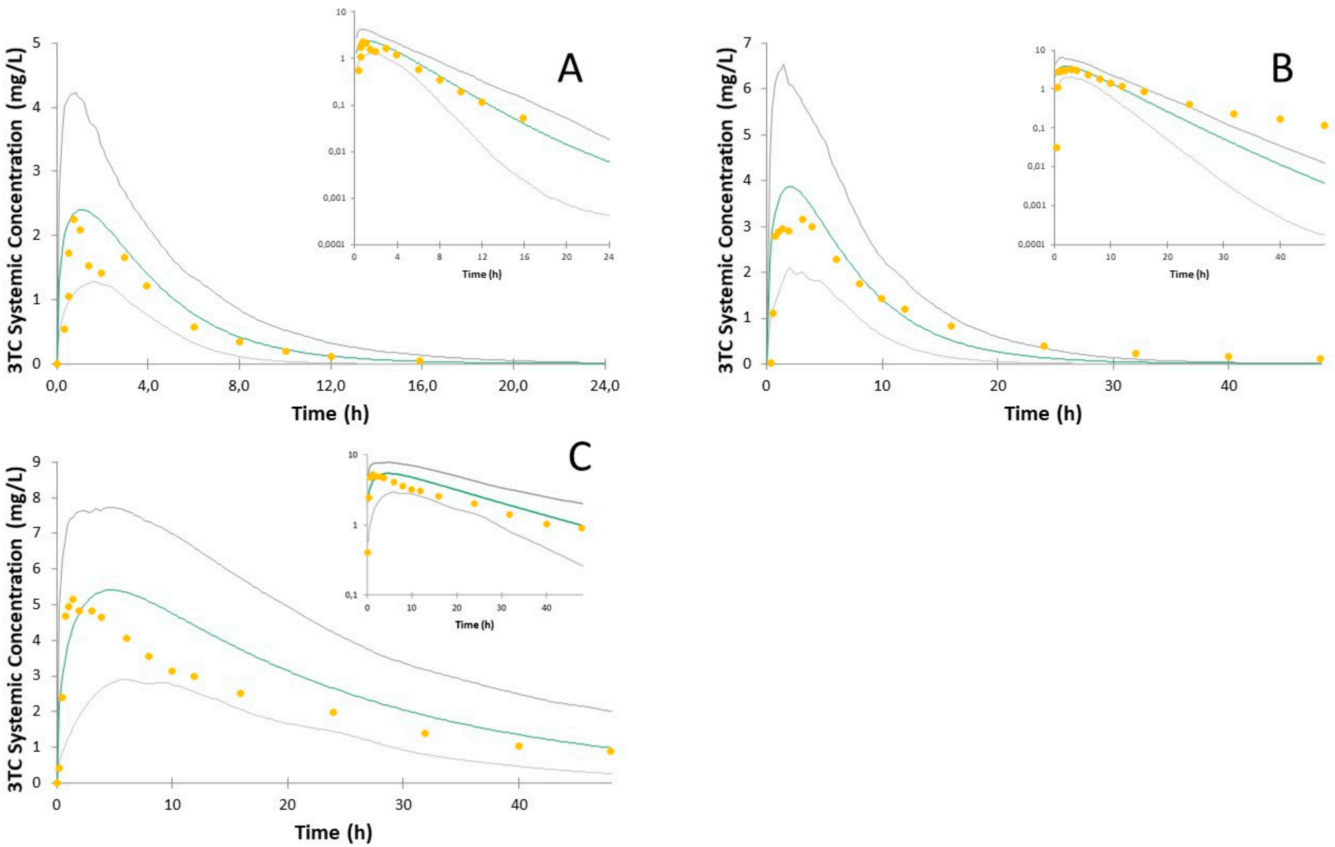

The green solid line is the predicted mean of the simulated population and the grey lines represent the 5th to 95th percentile around the predicted mean. Yellow circles are observed mean data from Heald et al.[6] Insets depict log-transformed plasma concentration–time data. **A**: Adults with normal kidney function receiving a single dose of 300 mg 3TC; **B**: Adults with GFR 10-40 mL/min/1.73m^2 receiving a single dose of 300 mg 3TC; **C**: Adults with GFR <10 mL/min/1.73m^2 receiving a single dose of 300 mg 3TC.

Figure S2.2 Visual predictive checks for prediction of 3TC PK in children with normal kidney function.

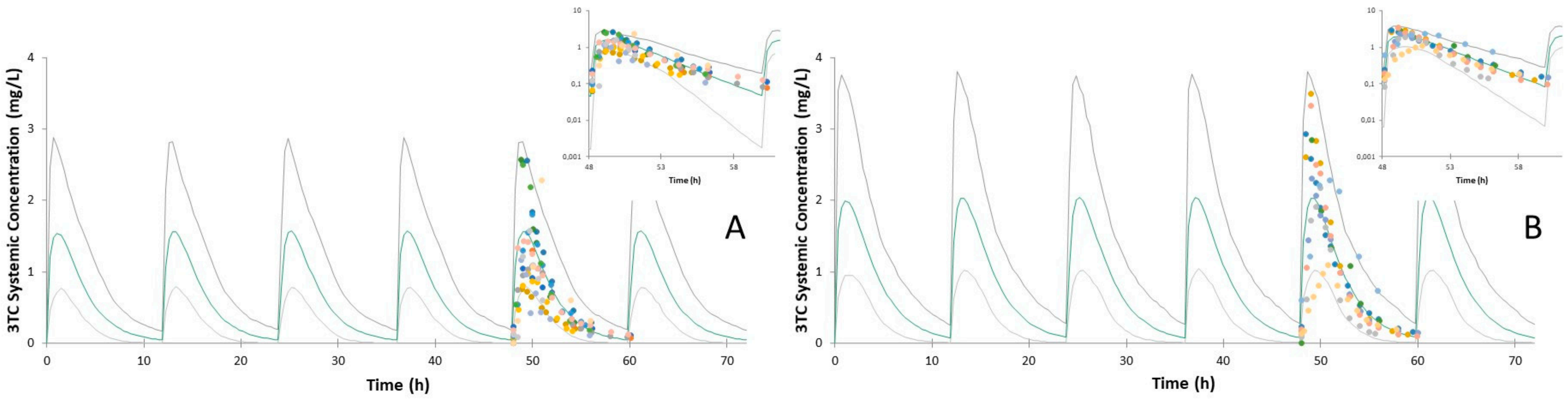

The green solid line is the predicted mean of the simulated population and the grey lines represent the 5th to 95th percentile around the predicted mean. Coloured circles are observed individual data from Burger et al.[4] Insets depict log-transformed plasma concentration–time data. **A**: Children weighing <25kg receiving multiple dosages of 4 mg/kg 3TC twice daily; **B**: Children weighing ≥25kg receiving multiple dosages of 150 mg 3TC twice daily.

Figure S2.3 Visual predictive checks for prediction of FTC PK in adults with various degrees of CKD.

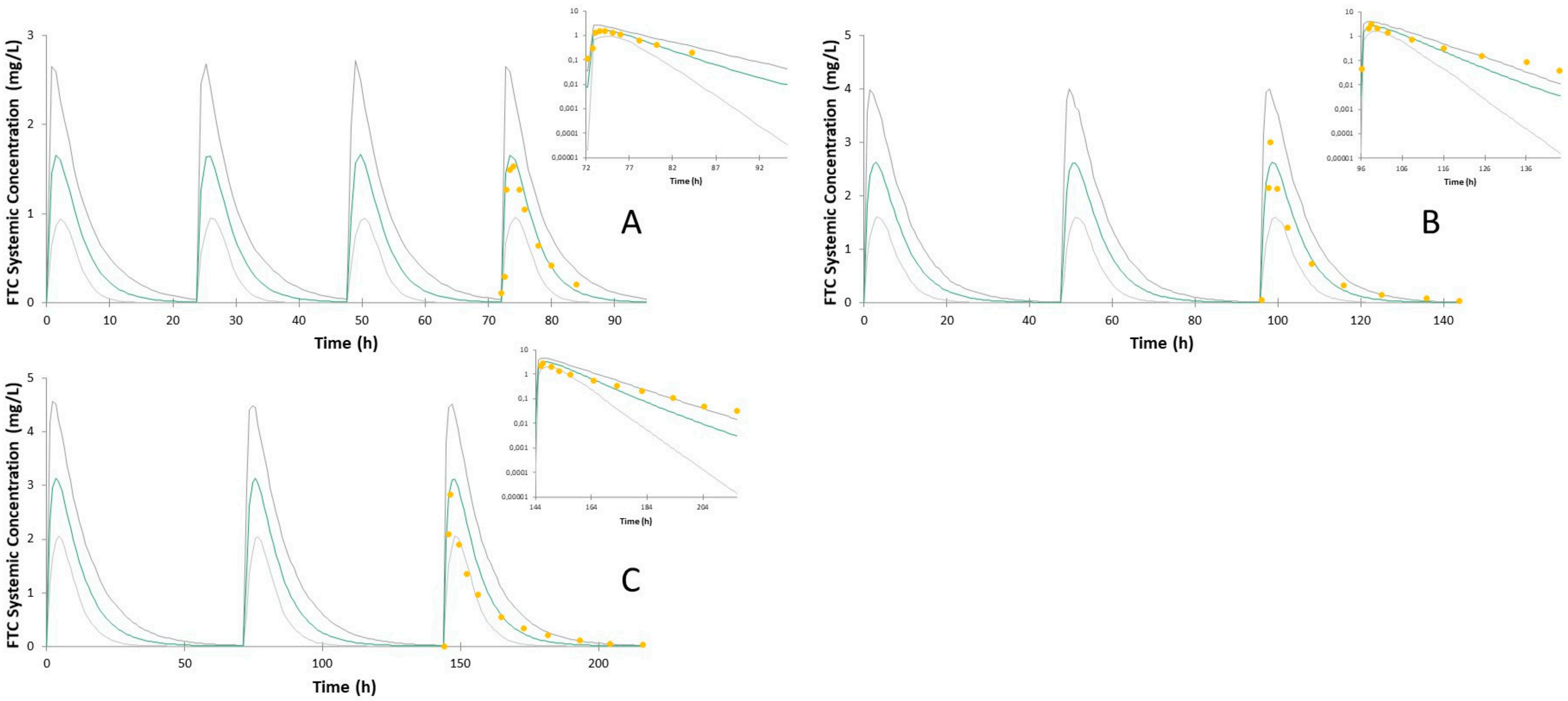

The green solid line is the predicted mean of the simulated population and the grey lines represent the 5th to 95th percentile around the predicted mean. Yellow circles are mean observed data from Wang et al. and the biopharmaceutical review of the FDA.[7,8] Insets depict log-transformed plasma concentration–time data**. A**: Adults with normal kidney function receiving multiple doses of 200 mg FTC once daily; **B**: Adults with GFR 30-50 mL/min/1.73m^2 receiving multiple doses of 200 mg FTC once every two days; **C**: Adults with GFR 15-30 mL/min/1.73m^2 receiving multiple doses of 200 mg FTC once every three days.

Figure S2.4 Visual predictive checks for prediction of FTC PK in children with normal kidney function.

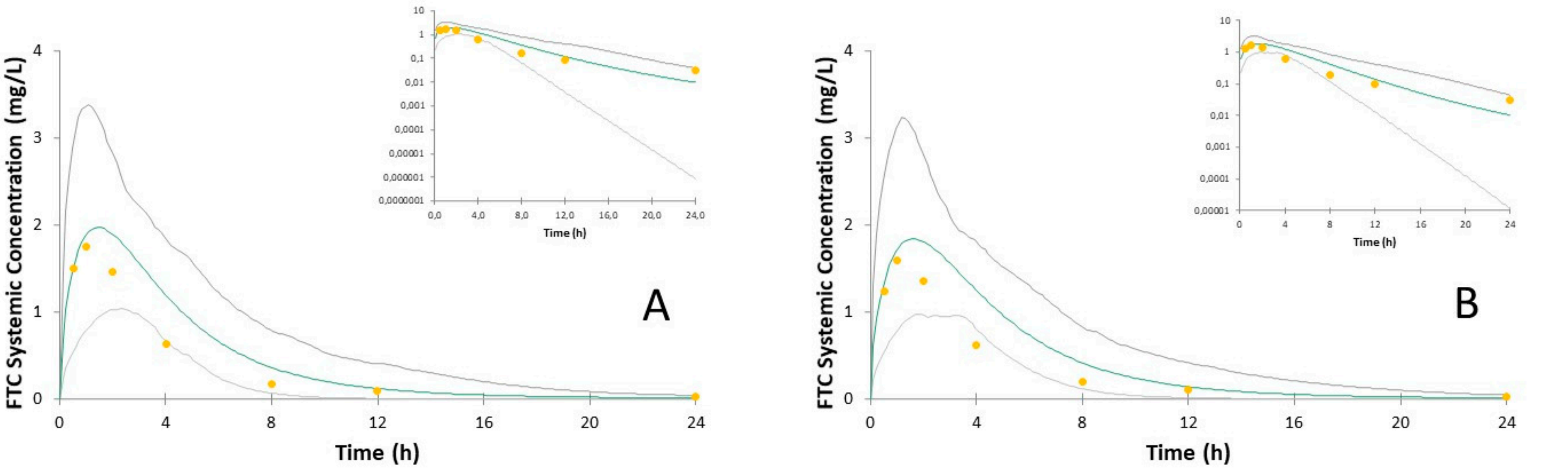

The green solid line is the predicted mean of the simulated population and the grey lines represent the 5th to 95th percentile around the predicted mean. Yellow circles are mean observed data from Wang et al.[9] Insets depict log-transformed plasma concentration–time data. **A**: Children <6 years old receiving a single dose of 120 mg/m^2 FTC; **B**: Children 6-12 years old receiving a single dose of 120 mg/m^2 FTC.

Table S9. Simulation results for 3TC PK in non-CKD and CKD populations.

Population	Kidney function	Type of dose	3TC dose	Dosing interval (τ)	Predicted $AUC_{0-\tau}$ (h·mg/L)	Ratio AUC_{0-24h}^{*}	Simulated C_{max} (mg/L)	Ratio C_{max}^{*}
Children 3 months – <2 years	No CKD	Standard dose	5 mg/kg	12h	9.73	1.00	2.25	1.00
	CKD G3	Standard dose	5 mg/kg	12h	22.02	2.26	3.60	1.60
		GFR-adjusted dose	2.5 mg/kg	12h	11.01	1.13	1.80	0.80
	CKD G4**	Standard dose	5 mg/kg	12h	33.91	3.49	4.64	2.06
		GFR-adjusted dose	2.5 mg/kg loading, then 1.88 mg/kg	24h	12.75	1.31	1.75	0.78
Children 2 – <6 years	No CKD	Standard dose	10 mg/kg	24h	19.62	1.00	4.52	1.00
	CKD G3	Standard dose	10 mg/kg	24h	45.02	2.29	6.89	1.52
		GFR-adjusted dose	5 mg/kg	24h	22.77	1.16	3.48	0.77
	CKD G4**	Standard dose	10 mg/kg	24h	71.32	3.64	8.42	1.86
		GFR-adjusted dose	5.0 mg/kg loading, then 3.25 mg/kg	24h	23.19	1.18	2.74	0.61
Children 6 – <12 years	No CKD	Standard dose	300 mg	24h	19.58	1.00	4.11	1.00
	CKD G3	Standard dose	300 mg	24h	44.79	2.29	6.10	1.48
		GFR-adjusted dose	150 mg	24h	22.40	1.14	3.05	0.74
	CKD G4**	Standard dose	300 mg	24h	72.35	3.70	7.49	1.82
		GFR-adjusted dose	150 mg loading, then 100 mg/kg	24h	24.14	1.23	2.50	0.61

Children 12 – <15 years	No CKD	Standard dose	300 mg	24h	15.95	1.00	3.34	1.00
	CKD G3	Standard dose	300 mg	24h	37.23	2.33	5.03	1.50
		GFR-adjusted dose	150 mg	24h	18.61	1.17	2.51	0.75
	CKD G4**	Standard dose	300 mg	24h	57.28	3.59	6.03	1.80
		GFR-adjusted dose	150 mg loading, then 100 mg/kg	24h	19.10	1.20	2.01	0.60
Adults	No CKD	Standard dose	300 mg	24h	11.96	1.00	2.36	1.00
	CKD G3	Standard dose	300 mg	24h	30.98	2.59	3.76	1.59
		GFR-adjusted dose	150 mg	24h	15.49	1.30	1.88	0.80
	CKD G4	Standard dose	300 mg	24h	48.43	4.05	4.67	1.98
		GFR-adjusted dose	150 mg loading, then 100 mg/kg	24h	16.17	1.35	1.56	0.66

Parameter values are reported as geometric means.
* Ratio: CKD population / no CKD population
** It should be noted that model performance was not verified for this population due the absence of clinical data.
To calculate $AUC_{0-\tau}$ ratios, dosing intervals should be similar. In case of discrepancy, the AUC corresponding to the shortest dosing interval is scaled to the highest dosing interval (e.g. AUC_{0-12h} times 2 to get AUC_{0-24h})
Abbreviations: 3TC, lamivudine; AUC, area-under-curve; BSA, body surface area; C_{max}, maximum concentration; fa, fraction of a dose absorbed; CKD, chronic kidney disease; G, grade; τ, dosing interval.

Table S10. Simulation results for FTC PK in non-CKD and CKD populations.

Population	Kidney function	Type of dose	FTC dose	Dosing interval (τ)	Predicted $AUC_{0-\tau}$ (h·mg/L)	Ratio AUC_{0-24h}*	Simulated C_{max} (mg/L)	Ratio C_{max}*
Children 3 months – <2 years	No CKD	Standard dose	6 mg/kg	24h	11.99	1.00	2.27	1.00
	CKD G3	Standard dose	6 mg/kg	24h	28.32	2.36	3.58	1.58
		GFR-adjusted dose	3 mg/kg	24h	14.16	1.18	1.79	0.79
	CKD G4**	Standard dose	6 mg/kg	24h	44.89	3.74	4.45	1.96
		GFR-adjusted dose	2 mg/kg	24h	14.96	1.25	1.48	0.65
Children 2 – <6 years	No CKD	Standard dose	6 mg/kg	24h	12.14	1.00	2.33	1.00
	CKD G3	Standard dose	6 mg/kg	24h	29.25	2.41	3.73	1.60
		GFR-adjusted dose	3 mg/kg	24h	14.62	1.20	1.87	0.80
	CKD G4**	Standard dose	6 mg/kg	24h	47.06	3.88	4.67	2.00
		GFR-adjusted dose	2 mg/kg	24h	15.69	1.29	1.56	0.67
Children 6 – <12 years	No CKD	Standard dose	200 mg	24h	15.28	1.00	2.62	1.00
	CKD G3	Standard dose	200 mg	24h	36.76	2.41	4.12	1.57
		GFR-adjusted dose	200 mg	48h	36.76	1.20	4.04	1.54
	CKD G4**	Standard dose	200 mg	24h	61.47	4.02	5.28	2.01
		GFR-adjusted dose	200 mg	72h	61.47	1.34	4.93	1.88
Children	No CKD	Standard dose	200 mg	24h	13.13	1.00	2.31	1.00

12 – <15 years	CKD G3	Standard dose	200 mg	24h	31.55	2.40	3.64	1.58
		GFR-adjusted dose	200 mg	48h	31.55	1.20	3.59	1.55
	CKD G4**	Standard dose	200 mg	24h	51.16	3.90	4.58	1.98
		GFR-adjusted dose	200 mg	72h	51.17	1.30	4.34	1.88
Adults	No CKD	Standard dose	200 mg	24h	9.68	1.00	1.60	1.00
	CKD G3	Standard dose	200 mg	24h	27.80	2.87	2.81	1.76
		GFR-adjusted dose	200 mg	48h	27.80	1.44	2.71	1.70
	CKD G4	Standard dose	200 mg	24h	44.23	4.57	3.62	2.26
		GFR-adjusted dose	200 mg	72h	44.23	1.52	3.30	2.06

Parameters reported as geometric means.

* Ratio: CKD population / no CKD population

** It should be noted that due the absence of clinical data, model performance was not verified for this population.

To calculate $AUC_{0\text{-}\tau}$ ratios, dosing intervals should be similar. In case of discrepancy, the AUC corresponding to the shortest dosing interval is scaled to the highest dosing interval (e.g. $AUC_{0\text{-}24h}$ times 3 to get $AUC_{0\text{-}72h}$)

Abbreviations: AUC, area under curve; BSA, body surface area; C_{max}, maximum concentration; fa, fraction of a dose absorbed; FTC, emtricitabine; CKD, chronic kidney disease; G, grade; τ, dosing interval.

References

1. Shah, K.; Fischetti, B.; Cha, A.; Taft, D.R. Using PBPK Modeling to Predict Drug Exposure and Support Dosage Adjustments in Patients With Renal Impairment: An Example with Lamivudine. *Curr Drug Discov Technol* **2020**, *17*, 387-396, doi:10.2174/1570163816666190214164916.
2. De Sousa Mendes, M.; Hirt, D.; Urien, S.; Valade, E.; Bouazza, N.; Foissac, F.; Blanche, S.; Treluyer, J.M.; Benaboud, S. Physiologically-based pharmacokinetic modeling of renally excreted antiretroviral drugs in pregnant women. *Br J Clin Pharmacol* **2015**, *80*, 1031-1041, doi:10.1111/bcp.12685.
3. Hsueh, C.H.; Hsu, V.; Zhao, P.; Zhang, L.; Giacomini, K.M.; Huang, S.M. PBPK Modeling of the Effect of Reduced Kidney Function on the Pharmacokinetics of Drugs Excreted Renally by Organic Anion Transporters. *Clin Pharmacol Ther* **2018**, *103*, 485-492, doi:10.1002/cpt.750.
4. Burger, D.M.; Verweel, G.; Rakhmanina, N.; Verwey-Van Wissen, C.P.; La Porte, C.J.; Bergshoeff, A.S.; Lyall, H.; Hartwig, N.G.; Green, H.; Soldin, S.; et al. Age-dependent pharmacokinetics of lamivudine in HIV-infected children. *Clin Pharmacol Ther* **2007**, *81*, 517-520, doi:10.1038/sj.clpt.6100118.
5. Johnson, M.A.; Verpooten, G.A.; Daniel, M.J.; Plumb, R.; Moss, J.; Van Caesbroeck, D.; De Broe, M.E. Single dose pharmacokinetics of lamivudine in subjects with impaired renal function and the effect of haemodialysis. *Br J Clin Pharmacol* **1998**, *46*, 21-27, doi:10.1046/j.1365-2125.1998.00044.x.
6. Heald, A.E.; Hsyu, P.H.; Yuen, G.J.; Robinson, P.; Mydlow, P.; Bartlett, J.A. Pharmacokinetics of lamivudine in human immunodeficiency virus-infected patients with renal dysfunction. *Antimicrob Agents Chemother* **1996**, *40*, 1514-1519, doi:10.1128/aac.40.6.1514.
7. Emtriva. Available online: https://www.accessdata.fda.gov/drugsatfda_docs/label/2008/021500s010,021896s004lbl.pdf (accessed on 22-01).
8. Wang, L.H.; Begley, J.; St Claire, R.L., 3rd; Harris, J.; Wakeford, C.; Rousseau, F.S. Pharmacokinetic and pharmacodynamic characteristics of emtricitabine support its once daily dosing for the treatment of HIV infection. *AIDS Res Hum Retroviruses* **2004**, *20*, 1173-1182, doi:10.1089/aid.2004.20.1173.
9. Wang, L.H.; Wiznia, A.A.; Rathore, M.H.; Chittick, G.E.; Bakshi, S.S.; Emmanuel, P.J.; Flynn, P.M. Pharmacokinetics and safety of single oral doses of emtricitabine in human immunodeficiency virus-infected children. *Antimicrob Agents Chemother* **2004**, *48*, 183-191, doi:10.1128/aac.48.1.183-191.2004.
10. Czock, D.; Scholle, C.; Rasche, F.M.; Schaarschmidt, D.; Keller, F. Pharmacokinetics of valganciclovir and ganciclovir in renal impairment. *Clin Pharmacol Ther* **2002**, *72*, 142-150, doi:10.1067/mcp.2002.126306.
11. Vaudry, W.; Ettenger, R.; Jara, P.; Varela-Fascinetto, G.; Bouw, M.R.; Ives, J.; Walker, R. Valganciclovir dosing according to body surface area and renal function in pediatric solid organ transplant recipients. *Am J Transplant* **2009**, *9*, 636-643, doi:10.1111/j.1600-6143.2008.02528.x.
12. Gaur, A.H.; Cotton, M.F.; Rodriguez, C.A.; McGrath, E.J.; Helström, E.; Liberty, A.; Natukunda, E.; Kosalaraksa, P.; Chokephaibulkit, K.; Maxwell, H.; et al. Fixed-dose combination bictegravir, emtricitabine, and tenofovir alafenamide in adolescents and children with HIV: week 48 results of a single-arm, open-label, multicentre, phase 2/3 trial. *Lancet Child Adolesc Health* **2021**, *5*, 642-651, doi:10.1016/s2352-4642(21)00165-6.
